# The Role of Fascia Iliaca Blocks in Hip Fractures: A Prospective Case-Control Study and Feasibility Assessment of a Junior-Doctor-Delivered Service

**DOI:** 10.1155/2014/191306

**Published:** 2014-03-04

**Authors:** L. Hanna, A. Gulati, A. Graham

**Affiliations:** Trauma and Orthopaedic Department, Stoke Mandeville Hospital, Mandeville Road, Aylesbury, Buckinghamshire HP21 8AL, UK

## Abstract

Hip fractures are common and the incidence is expected to increase. Systemic analgesics, often prescribed to relieve pain after hip fractures, have huge side effects and can delay surgery. We analyse the role and efficacy of alternative forms of analgesia like fascia-iliac blocks (FIB) and assess the feasibility of a service delivered by junior doctors. 104 consecutive hip fracture patients were prospectively recruited and equally divided into cases (patients receiving FIB) and controls (patients receiving systemic analgesia). Outcome measures included time of initial analgesia, total preoperative dose of analgesia, pain scores from admission to 24 hours preoperatively, and complications. The pain scores were significantly lower (*P* ≤ 0.05) in patients receiving FIB at 2 and 8 hours preoperatively. The timing of initial analgesia was also quicker in patients with FIB (25 compared to 40 minutes). FIB patients required fewer doses of systemic analgesia. The block was successful in 67% of patients. There were no complications. The implementation of EWTD, HAN, and shift-system and the reduction in the number of medical staff have increased the burden on emergency departments. This study demonstrates that FIB performed by junior doctors are not only safe and effective analgesia but also provide an opportunity for junior doctors to improve current clinical practice.

## 1. Introduction

About 75,000 hip fractures occur annually in the United Kingdom [1] often as the result of trivial injury. The incidence of these “fragility fractures” is expected to increase to 91,500 by 2015 and 101,000 in 2020 [2] with people >85 years which are 10–15 times more likely to sustain hip fractures than people aged 60 to 65 years [3]. The seriousness of hip fractures is reflected by the 23-day postoperative hospital stay and 10% 30-day mortality, associated with an annual cost of medical and social care amounting to nearly *£*2 billion. These figures are multifactorial and are in part due to the complications that occur after a hip fracture in a population group with significant comorbidities (median ASA grade 3) [4].

Hip fractures are painful and inadequately controlled pain can have significant physiological and psychological effects [5] such as an acute confusional state (delirium) seen in 10–16% of hip fracture patients presenting to emergency department (ED) [6]. These factors make further pain assessment difficult and have the potential to delay surgical intervention, compound complications, and ultimately prolong hospital stay and increase the risk of nursing home placement [7]. The delivery of effective pain relief for hip fracture patients at the first point of contact in the emergency department (ED) is therefore crucial. Beside this, many studies suggest that pain management for limb fractures in the elderly is poor with as little as 2% of patients receiving adequate analgesia [8–10].

Systemic analgesia including both opioids and nonsteroidal analgesia can have significant adverse effects especially in the elderly population due to age-related changes in pharmacokinetics and pharmacodynamics [11]. Furthermore, the long list of medications that accompany most patients also increases the risk of drug interactions. A lack of in depth knowledge about such issues and how to manage them by ED doctors may further hinder effective analgesia prescribing [12].

Fascia iliaca blocks (FIBs) provide regional pain relief and can address many of the issues surrounding traditional forms of analgesia. They have traditionally been a remit of anaesthetic professionals perioperatively targeting the lateral cutaneous, femoral, and obturator nerves found beneath the fascia iliac. While doctors in other specialities, such as the ED, have carried out the blocks, routine administration in clinical practice has been staggered with only 19% of patients receiving a peripheral nerve blockade for hip fracture [4]. The reason for this is not known but could be due to concerns over adequate training and the misconception that administration takes longer than systemic analgesia.

This study describes our experience of FIB applied in the emergency setting and compares outcomes to traditional systemic analgesia. In addition, we demonstrate the feasibility of junior orthopaedic and emergency doctors administering this form of pain relief at the point of need.

## 2. Material and Methods

### 2.1. Study Type and Setting

A prospective case-control study was carried out over a six-month period in a large UK district general hospital involving the orthopaedic and emergency departments. This study was approved by the local audit committee.

### 2.2. Patient Selection

The patients were divided into two groups after informed verbal consent.

All patients with hip fractures presenting to the ED from January to March 2012 received traditional forms of systemic analgesia according to the WHO pain ladder (controls).

All patients with hip fractures presenting to the ED from April to June 2012 received FIB (cases).

The absolute contraindications to FIB included patient refusal, bleeding diatheses, femoral grafts in the affected limb, and inflammation over the injection site and allergy to local anaesthetics.

### 2.3. Fascia Iliac Block Training

All junior doctors (foundation year 2—core training year 2) in the trauma and orthopaedics department were trained on administering FIB through a teaching presentation from an anaesthetic trainee, followed by supervised training in theatre by a senior anaesthetist. Once deemed competent by the consultant anaesthetist, juniors were permitted to administer a single FIB per patient during the day and night on-call period. Once a robust orthopaedic service delivery was in place, ED junior doctors were also trained and administered blocks allowing for an earlier use of the block. After receiving the block, codeine and paracetamol were prescribed on the “as-required” section of the drug chart. Regular systemic analgesia including paracetamol and opioids was commenced if operative intervention was delayed by more than 24 hours, if required.

### 2.4. Fascia Iliac Block Technique

**Figure 1:**
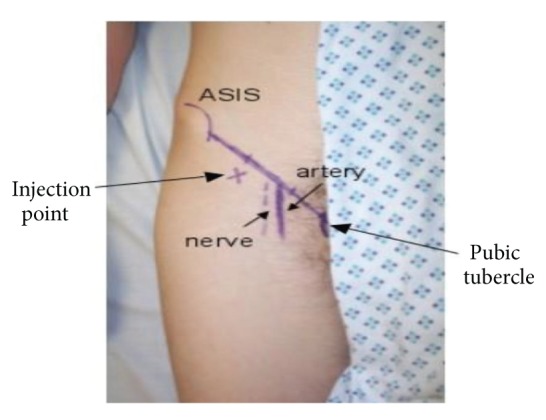
Injection site was located along the lateral one-third of a line joining the ASIS and PT targeting the compartment between fascia iliaca and fascia lata.

An 18 G Tuohy needle was used to administer a weight-dependant volume of 0.25% chirocaine (levobupivicaine) as per local anaesthetic protocol (Table 1) under aseptic technique. The injection site was located along the lateral one-third of a line joining the anterior superior iliac spine (ASIS) and pubic tubercle (PT) targeting the compartment between fascia iliaca and fascia lata (Figure 1).

### 2.5. Outcome Measures

Demographic data (age, sex), Abbreviated mental test score (AMTS) and fracture classification (intracapsular/extracapsular) was recorded (Table 2). Pain scores were measured on gentle pin rolling of the affected leg in both groups and assessed through a visual analogue scale of 0–10 preanalgesia/block and then at 15 minutes, 2, 8, 16, and 24 hours after analgesia/block. At *T* = 15 mins, monitoring was carried out by the clinician who performed the block and also included monitoring of blood pressure, heart rate, and oxygen saturation. Thereafter, monitoring was carried out in orthopaedic wards by healthcare professionals who received appropriate education and training about the project and how to elicit pain scores and their documentation. Block success was deemed as pain score <50% to the preblock score at 2 hours after block administration as per the standard of care agreed by the National Collaborative that a pain score should never exceed 50 per cent [13, 14]. The time of initial analgesia, total preoperative dose of analgesia required, and any complications were also recorded.

### 2.6. Analysis

Analyses were carried out using the statistical package SPSS for Windows (V.18.0, Chicago, Il, USA). For continuous data, Student's *t*-tests were used for two groups of variables and one-way analyses of variance (ANOVA) for more than two groups, followed by post-hoc Tukey analysis. For categorical data, Pearson's Chi-square test was used with statistical significance reached by a *P* value of <0.05.

## 3. Results

### 3.1. Demographics

From January 2012 to June 2012, a total of 104 patients were included in the study. 52 received systemic analgesia as per the traditional methods (controls) and 52 patients received a FIB as soon as possible after radiological diagnosis (cases). Four patients were excluded from receiving a FIB, 3 due to a femoral graft in the affected limb and one due to severe aggressive dementia preventing safe administration. There were no complications and specifically no infections at the injection site. There were no wound infections postoperatively. The demographics of the two groups and their preoperative variables are shown in Table 2. Outcome measures are shown in Table 3.

## 4. Discussion

Hip fractures are painful and pain left untreated can result in a host of complications that may delay operative intervention and complicate hospital stay [15]. Pain management in many hospitals in the UK is based upon the use of systemic analgesia according to local hip fracture protocols. With more than 313000 patients waiting more than 4 hours in the ED before being seen [16] the busy nature of the ED and increased patient-to-staff ratio may delay pain assessment and treatment, with one study reporting mean time to pain assessment of 40 minutes and mean delay to treatment of 122 minutes [17]. The National Institute of Clinical Excellence guidelines suggest considering the use of neural blockade by trained personnel to limit opioid dosage [2].

This study demonstrates the beneficial effect of FIB, performed by junior doctors in ED, in management of pain in hip fracture patients. There was a significant reduction in pain scores at 2 hours (*P* = 0.03) following blockade which continued for up to 8 hours (*P* = 0.01). While this study did not find any significant difference at 15 min, two previous studies have reported reduced pain scores at 15 min after block [11, 18]. The pain scores were also reduced to half at 16 and 24 hours after block but they were not statistically significant (Table 3).

The time to initial analgesia was also reduced (25 mins versus 40 mins, *P* = 0.04) (Table 3) with most patients and relatives verbally reporting satisfaction with the prompt pain service delivery by the admitting clinician (no quantifiable data available). Systemic analgesic requirements were also significantly reduced within 24 hours of admission, a similar finding by Monzon et al. [19]. This rapid and long lasting effect of a single FIB makes this form of analgesia attractive in the busy surroundings of the emergency department and acute orthopaedic wards.

The beneficial effect of FIBs in patients with radiologically confirmed hip fractures is well known with several studies reporting a good outcome [11, 20–23], when compared to NSAIDS [11, 20], alfentanil [21], and placebo [22, 23]. Its success is attributable to blocking pain sensation in the femoral nerve, lateral cutaneous nerve of the thigh, and obturator nerve. Pain relief has been found to be both at rest and upon movement [18, 23]. This is beneficial in allowing patients to sit up more comfortably while they await surgery [24] and can facilitate spinal anaesthesia [21].

Mouzopoulos et al. [22] also found a reduction in the occurrence of delirium in hip fracture patients who have had a block, most likely as a result of the opioid sparing effect in this particularly vulnerable population group [23]. This was however not analysed in the current study.

Our block success was 65%, which is on the lower site of the reported rates of 67%–96% [12, 17, 23, 25]. This could be due to the fact that different authors have used different definitions for the block success, for instance, pain score reduction of <3 [25], sensory loss over the thigh [23], and an increase in flexion [18, 24]. We defined our block success as a 50% reduction in pain score at *T* = 2 hr from preblock pain score based on the suggestion by Counsell [13] and the NHS Fractured Neck of Femur Collaborative (NHS Modernisation Agency, 2001) [14].

There are a number of ways to administer the FIB such as the loss of resistance (“2-pop techqnique”) that we used, USS guided blocks, or the nerve stimulators to locate the femoral nerve. USS guided blocks are not practical for all EDs due to training, timing, and resource limitations. The use of nerve stimulators has been shown to be no better than the loss of resistance technique. In fact the time for peripheral nerve stimulator block was significantly longer [26].

While there are publications detailing the use of intradermal needles for the block, we used the blunt Tuohy needles which provide a further margin of safety and give a better “pop” sensation as the needles traverse the fascia, thereby reassuring the junior doctor that they are in the right compartment. As there are no reported studies comparing this technique, it would seem reasonable that this form of analgesia may be administered in the ED according to local resource and expertise available.

The main limitations of this study are that the patients were not randomised, although the two groups were comparable (Table 2). Furthermore, some patients were given analgesia by the ambulance staff prior to presentation in the ED; however, this information was not always available. As a result this study might have underestimated the additional doses of analgesia required in some patients. Since the guidelines for analgesia prescription in suspected hip fracture patients have not changed for the ambulance staff, it is quite possible that the two groups were also comparable for this variable.

The implementation of European Working Time Directive (EWTD), Hospital at Night, shift system has increased the burden on ED. As junior doctors are increasingly at the forefront of service delivery, administering FIBs is an essential skill to possess that can extend beyond anaesthetic training and the theatre environment. Furthermore, it encourages a more active role in pain management. This study demonstrates that the junior doctors without anaesthetic backgrounds can provide a rapid and effective pain service, supporting change to current clinical practice.

With only 71.4% of hip fracture patients receiving their operation within the recommended 36 hours and the majority of delays occurring due to medical causes including complications of pain management [27], the administration of FIB by junior doctors provides promising opportunity. Such a service has far-reaching effects and can also contribute to the provision of a streamlined high-quality perioperative hip fracture service that is necessary in the current climate of shift systems imposed by the EWTD. The use of continuous FIB using indwelling catheters, as piloted by Dulaney-Cripe et al. [28], may be of further benefit and is currently under ongoing investigation.

In addition, utilising the expertise of an existing anaesthetic department could facilitate adequate training. Not only will this be time and cost effective but also will foster integrated learning between surgical, emergency, and anaesthetic specialities.

## Figures and Tables

**Table 1:** Weight-dependant volumes used for FIB.

Weight	Volume of chirocaine (mLs)
<40 kg	20
40–80	30
>80 kg	40

**Table 2:** Demographics and fracture characteristics of patient groups.

	Controls *n* = 52	Cases *n* = 52	*P* value
Demographics			
Age	82 (49–95)	81 (25–100)	0.13
Male versus female	14 versus 38	19 versus 33	0.42
AMTS	8 (0–10)	9 (0–10)	0.52
Fracture characteristics			
Left side	22	23	0.78
Right side	30	29	0.82
Extracapsular	23	28	0.09
Intracapsular	29	24	0.08

**Table 3:** Pain scores, time to initial analgesia, and average number of additional doses of analgesia required.

	Controls *n* = 52	Cases *n* = 52	*P* value
Average pain scores (0–10)			
On admission/preblock	8	8	0.54
15 mins	6	6	0.11
2 hr	7	4	0.03
8 hr	7	3	0.01
16 hr	6	3	0.10
24 hr	6	3	0.13
Average time to initial analgesia (min)	40	25	0.04
Average number of doses of additional analgesia (range)	3.5 (0–7)	1.5 (0–3)	Not calculated
